# State of *Panax ginseng* Research: A Global Analysis

**DOI:** 10.3390/molecules22091518

**Published:** 2017-09-11

**Authors:** Wanqi Xu, Hyung-Kyoon Choi, Linfang Huang

**Affiliations:** 1 Institute of Medicinal Plant Development, Chinese Academy of Medical Sciences (CAMS), Peking Union Medical College (PUMC), Beijing 100193, China; wqxumio@163.com; 2 College of Pharmacy, Chung-Ang University, Seoul 156-756, Korea; hykychoi@cau.ac.kr

**Keywords:** *Panax ginseng*, bibliometrics, global cooperation, emerging trends

## Abstract

This article aims to understand the global and longitudinal trends of research on *Panax ginseng*. We used bibliometrics to analyze 3974 papers collected from the Web of Science^TM^ Core Collection database during 1959–2016. The number of publications showed a steady growth before 2000 and exponentially increased in stage III (2000–2016, about 86% of the papers were published). Research on *P. ginseng* was conducted in 64 countries, mainly in Asia; in particular, 41% and 28% of the publications were from South Korea and China, respectively. The institutions from South Korea and China had high publication output and close cooperation and provided the majority of financial support. All top 10 authors and four of the top 20 journals in terms of number of publications originated from South Korea. The leading research subjects were pharmacology (39%), plant science (26%), and integrative complementary medicine (19%). The hotspot of *P. ginseng* research transformed from basic science to application, and multidisciplinary sciences will play a substantial role in the future. This study provides a comprehensive analysis to elucidate the global distribution, collaboration patterns, and research trends in the *P. ginseng* domain.

## 1. Introduction

*Panax ginseng* is a perennial herb that belongs to the Araliaceae family and is distributed in 35 countries, mainly in Asia, particularly South Korea and China [1]. Since ancient times, *P. ginseng* has been used as traditional medicine because of its heart-protective [2], anticancer [3], and neuroprotective properties [4]. Since the 21st century, the number of publications on *P. ginseng* has exponentially increased; more than 3400 articles, including 242 reviews, have focused on *P. ginseng*. However, few studies on *P. ginseng* employed bibliometrics analysis. In 2010, Kim [5] provided an overview of the trends in ginseng research. Therefore, understanding *P. ginseng* research from the global and longitudinal perspectives is crucial.

Bibliometrics analysis can be used to delineate development trend of an academic research domain, explore current research emphasis and hotspot, and predict future research focus and achievement [6,7]. This analytical method is especially suitable for *P. ginseng* research, which is a complex and multidisciplinary research field evolving rapidly since 2000.

In this study, we perform a bibliometric analysis of global research on *P. ginseng* between 1959 and 2016. This paper aims to reveal the intellectual landscape of *P. ginseng* and identify cooperation patterns, significant authors and papers, and emerging trends.

## 2. Data Collection and Methods

Data were retrieved from the Web of Science^TM^ Core Collection database. “*Panax ginseng*” was chosen as the search topic, and the retrieval time span was set to 1900 to 2016. A total of 3974 records were obtained between 1959 and 2016. Every bibliographic record in SCI contains the author, title, source, abstract, keywords, and cited references of a study.

A total of 64 countries, 9612 authors, 999 journals, 6609 keywords, and 13 languages were counted by HistCite software, a tool used for literature and statistical analyses. The original records were visually analyzed using the information visualization software CiteSpace V, which was invented by Dr. Chen Chaomei from the Drexel University. CiteSpace is the most advanced and distinctive information visualization tool that can reveal the intellectual landscape and detect recent emerging trends.

## 3. Results

**Figure 1:**
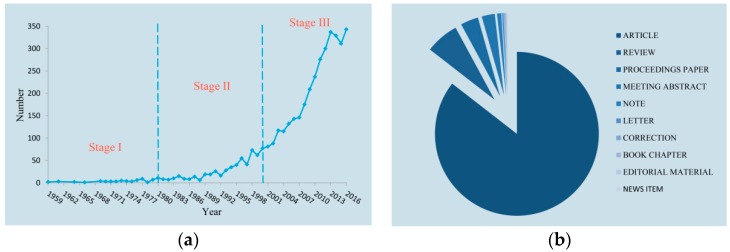
The status of publications. (**a**) Publication counts during 1959–2016 per year; (**b**) Types of publications.

Figure 1a shows the number of publications from 1959 to 2016. The number of published articles about *P. ginseng* generally increases annually. Since 1959, when German scientist PETKOV W [8,9] started to investigate the pharmacology and pharmacodynamics of *P. ginseng*, 3974 papers were published until 2016. Based on the number of publications, the past 60 years can be preliminarily divided into three stages, namely, stage I, 1959–1979; stage II, 1980–1999; and stage III, 2000–2016. Stage I (1959–1979) was considered the budding period, when less than 10 papers were published annually. Stage II (1980–1999), also known as the development period, began in 1980, when the number of annual publications reached 10. Stage III (2000–2016) or the boom period is the phase when an increased number of scholars began to focus on *P. ginseng* research.

According to publication category (Figure 1b), the 3974 publications obtained mainly included 3398 formal research articles (85.6%). A total of 262 review articles, 140 proceedings papers, and 103 meeting abstracts accounted for 6.6%, 3.5%, and 2.6% of the publications, respectively. Moreover, 33 notes, 13 letters, 7 corrections, 7 book chapters, 6 editorial materials, and 5 news items comprised less than 1.8% of all the publications.

**Figure 2:**
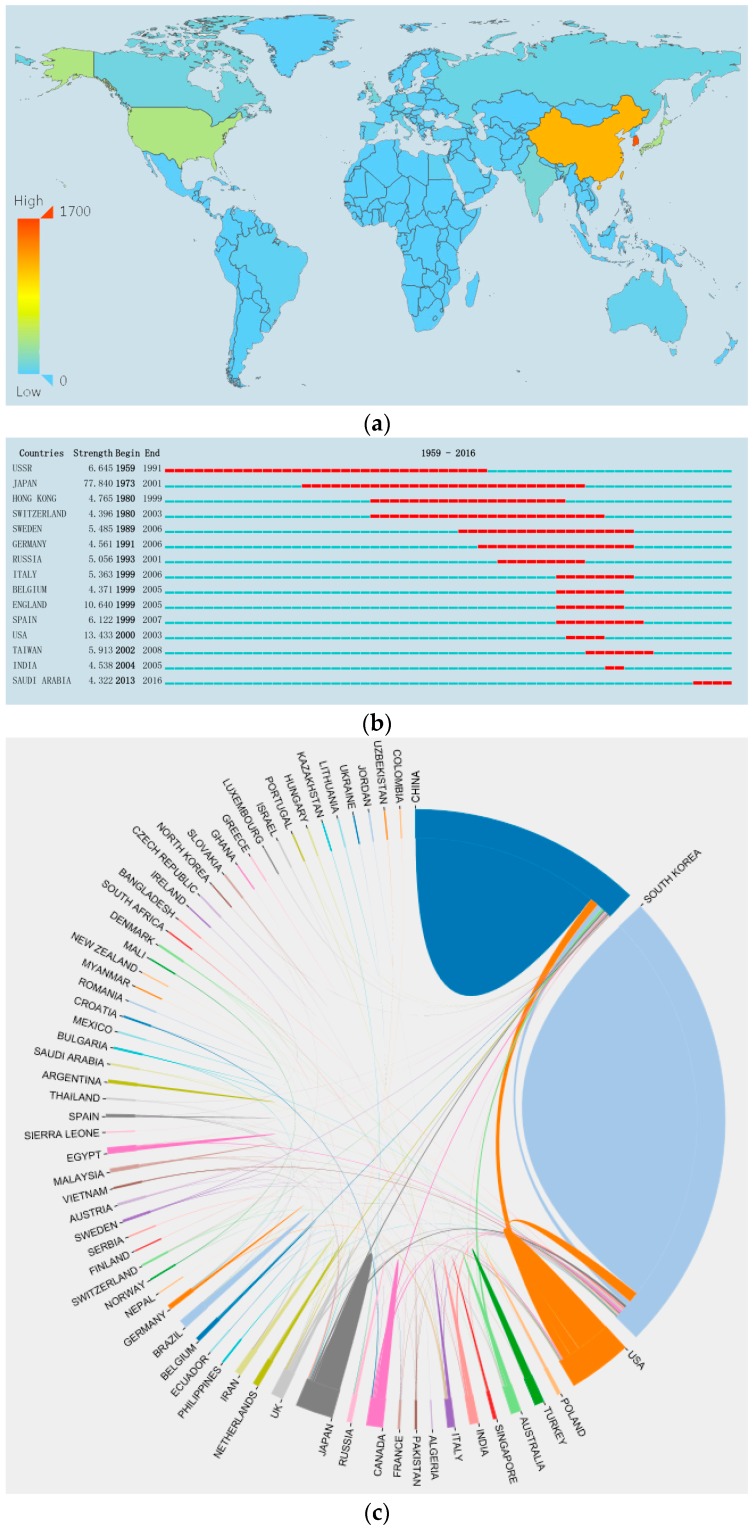
Global research status of *Panax ginseng*. (**a**) Geographical distribution of research countries; (**b**) 15 countries with burst detection among 64 countries; (**c**) Global cooperation.

Based on the heat map of the geographical distribution of research countries (Figure 2a), Asia, North America, and Europe produced the highest number of publications. *P. ginseng* research was conducted in 64 countries. In the first tier, South Korea ranked first in terms of research output by contributing 1632 articles (41.1%), and China ranked second with 1191 publications (27.5%). In the second tier, 396 papers originated from the USA and 381 papers from Japan. In the third tier, India, UK, Canada, and Russia published 127, 109, and 79 papers, respectively.

Burst detection is a computational technique used to identify abrupt changes in events and other types of information [10]. A burst is detected through two attributes, namely, strength and duration [11]. The red line segment of the column indicates the time period of burst detections. Figure 2b shows 15 countries with burst detection during 1959–2016. Of these countries, Japan exhibited the highest strength of 77.84 from 1973 to 2001. Hence, Japan conducted substantial works on *P. ginseng* during these years. Scholars from Saudi Arabia started showing interest in ginseng research from 2013 and contributed 13 publications.

As shown in Figure 2c, the top four countries (South Korea, China, USA, and Japan) with the highest publication number worked in close cooperation with one another. These four countries also worked closely with Canada, Germany, Norway, UK, Australia, Egypt, France, Italy, and India.

Table 1 lists the top 10 organizations that conducted and provided funds for *P. ginseng* research. Of these organizations, eight originated from South Korea and two, namely, Chinese Academy of Sciences and Jilin University, were from China. Similarly, the top 10 sponsor organizations comprised 70% Korean institutions and 30% Chinese institutions (National Natural Science Foundation of China, Ministry of Science and Technology of China, Fundamental Research Funds for the Central Universities). The National Natural Science Foundation of China funded for 263 papers, accounting for almost half of the articles funded by the top 10 sponsor organizations.

**Figure 3:**
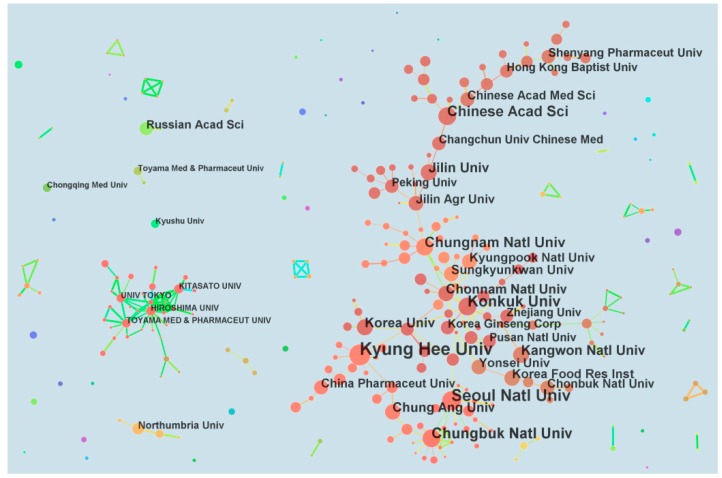
The collaboration patterns of global research institutions.

Figure 3 shows the cooperation among global research institutions. The thick line indicates high collaboration frequency. A linear partnership was found among Chinese institutions such as Shenyang Pharmaceutical University, Hong Kong Baptist University, Chinese Academy of Medical Sciences, Chinese Academy of Sciences, Changchun University of Chinese Medicine, Jilin University, and Jilin Agricultural University. Numerous Korean institutions presented group cooperation relationship to Jilin Agricultural University from China. Kyung Hee University, Chungbuk National University, Seoul National University, and Konkuk University were in the center position. These Korean institutions cooperated with Zhejiang University and China Pharmaceutical University from China. In Japan, most research institutions exhibited strong cooperation with one another. Five organizations (Russian Academy of Sciences, Toyama Medicinal and Pharmaceutical University, Chongqing Pharmaceutical University, Kyushu University, and Northumbria University) conducted *P. ginseng* research individually rather than cooperating with other institutions.

**Figure 4:**
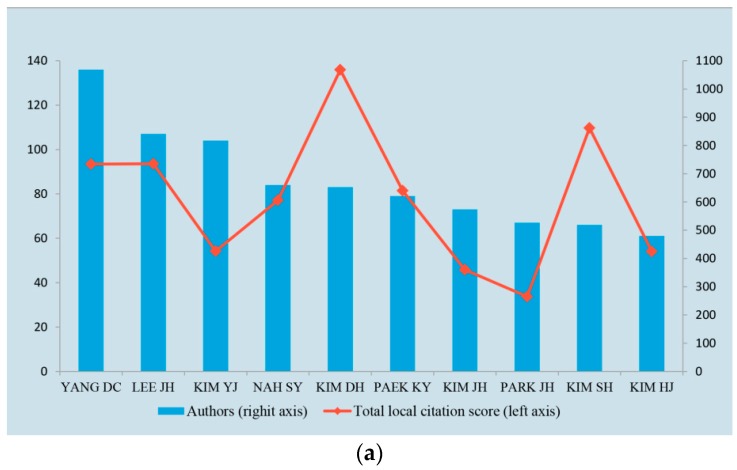

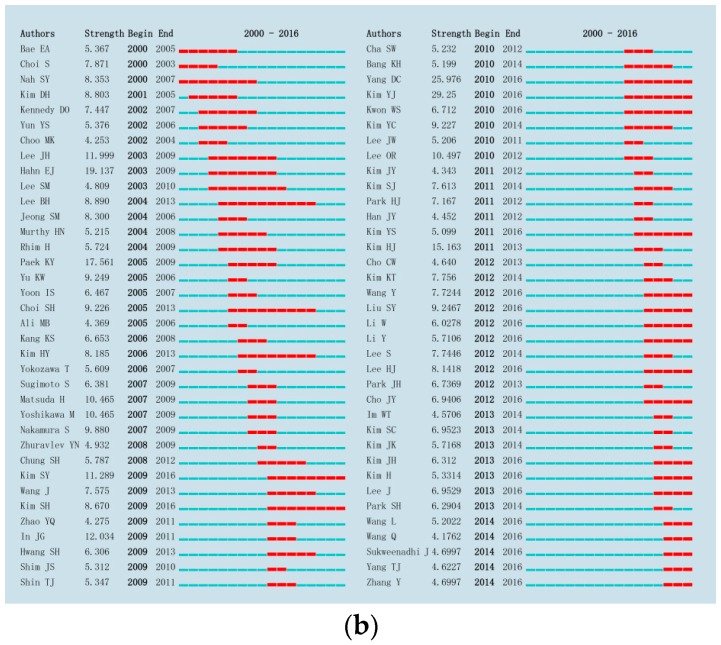
Authors with highest publication numbers and burst detection result. (**a**) Top 10 highest publishing authors and total local citation score; (**b**) Authors with strongest burst since 2000.

Figure 4a shows the publication outputs of top 10 authors and their total local citation scores. The top 10 authors originated from Korean institutions; in particular KIM DH and KIM SH contributed the highest number of publications and had the highest citation rate. Basing on the exponential increase in publication numbers during 2000–2016, we focused on the authors who started to burst from 2000 (Figure 4b). Twenty authors, including YANG DC, KIM YJ, KIM JH, and KIM SH, who also belong to the top 10 authors, had bursts from different years until 2016. Particularly, the articles of KIM SH showed high citation rate. In addition, Yuan CS, Wu JA, and Attele AS from University of Chicago had the highest citation rate and published 29, 7, and 3 articles, with TLCS of 1115, 765, and 684, respectively.

**Figure 5:**
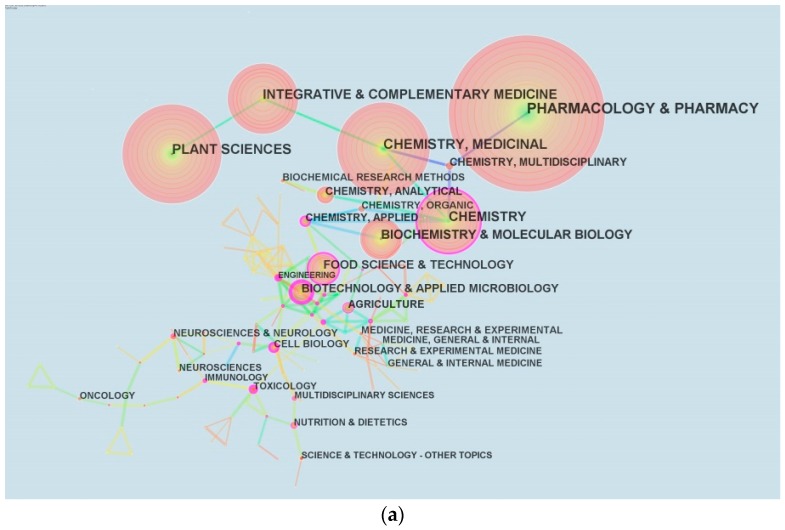

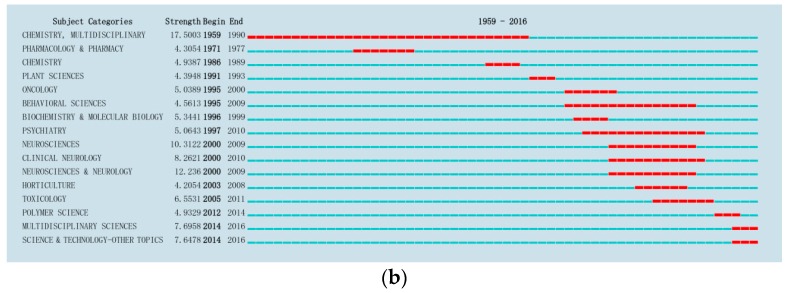
Major disciplines of *P. ginseng* and burst detection result. (**a**) Category co-occurrence network; (**b**) 16 subject categories have occurrence burst during 1959–2016.

Each paper indexed by the Web of Science^TM^ Core Collection was assigned with one or more subjects. A total of 120 unique subject categories were found (Figure 5a). The most common category (presented with the largest circle) is pharmacology and pharmacy, followed by plant science, chemistry, and integrative and complementary medicine. The nodes with thick purple ring have high betweenness centrality, which represents great transformation potential of a scientific contribution, and values tend to identify the boundary spanning potential that could lead to transformative discoveries [10,11]. Although engineering, biotechnology and applied microbiology, and toxicology and cell biology occupy a small space, their rings in purple indicate high betweenness centrality.

The subject categories of the included papers were analyzed to determine their burstness (Figure 5b). Sixteen subject categories were detected with bursts. Multidisciplinary sciences, science and technology, and other topics showed burst from 2014 to 2016.

**Figure 6:**
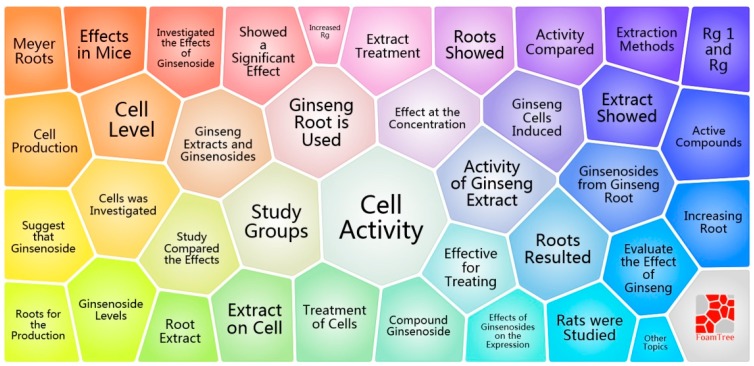
A visual survey of major topics on *P. ginseng* generated by the Carrot system.

The major topics in *P. ginseng* research are shown in Figure 6. The visual representation, known as a form tree, was generated using clustering software Carrot based on the 38 clusters of the 3974 publications. The leading topics in *P. ginseng* research are cell activity, activity of the ginseng extract, study groups, use of ginseng root, cell investigation, induction of ginseng cells, and treatment of cells.

**Figure 7:**
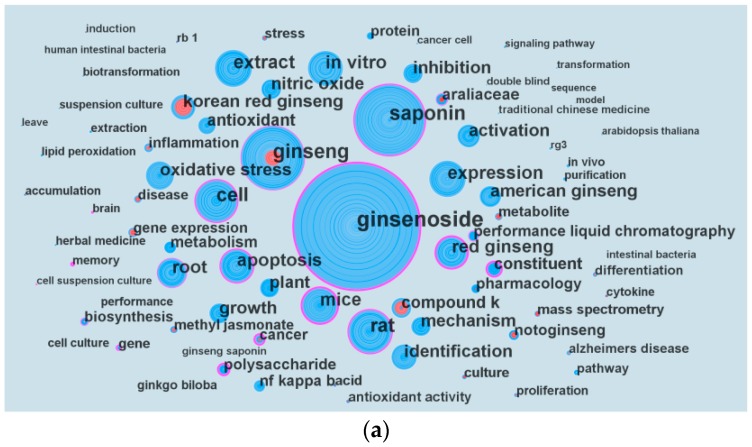

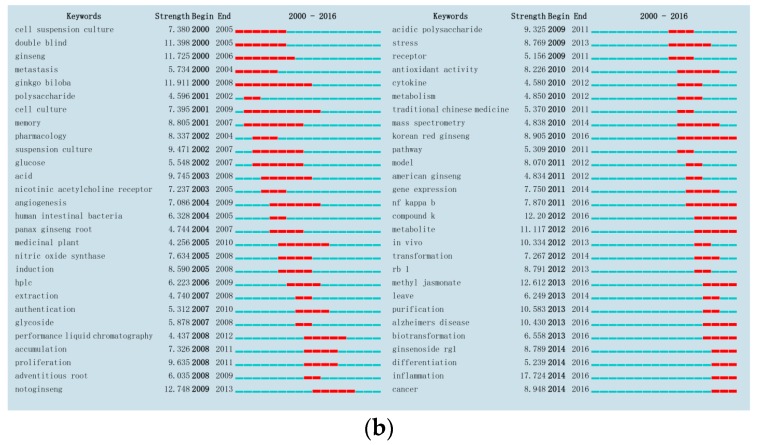
Major keywords of *P. ginseng* research and burst detection result. (**a**) Major keywords on *P*. *ginseng* research; (**b**) Keywords with strongest frequency burst since 2000.

Figure 7a shows the major keywords of *P. ginseng* research. The top 10 keywords in terms of the frequency of occurrence are: ginsenoside, saponin, ginseng, rat, cell, extract, mice, expression, in vitro, and apoptosis. The purple rings of ginsenoside, saponin, constituent, cell, red ginseng, rat, mice, apoptosis, cancer, and polysaccharide indicate their betweenness centrality, and the red ring indicates burst. This representation reveals the development of *P. ginseng* research focus (Figure 2b).

At a fine-grained level, keywords with burst reveal the new trend in *P. ginseng* research. Seventy-seven keywords showed burst. Considering that many articles were published after 2000, we focused on keywords with burst since 2000 (Figure 2b). The burst of keywords until 2016 are Korean red ginseng, nf kappa b, compound k, metabolite, methyl jasmonate, Alzheimer’s disease, biotransformation, ginsenoside rg1, differentiation, inflammation, and cancer. Inflammation, methyl jasmonate, compound k, metabolite, and Alzheimer’s disease had the strongest burst strengths of over 10.

A total of 3974 papers were found in 999 different journals. Table 2 shows that approximately all of the papers were written in English (98.0%), and the remaining papers were written in 12 different languages, such as Chinese (34), Japanese (16), and Russian (11). A few papers were written in Portuguese, German, Polish, French, Hungarian, Italian, Korean, Spanish, and Turkish.

Table 3 displays that most of the top 20 highest publishing journals originated from South Korea (4), Germany (3), England (3), the USA (3), and the Netherlands (3).

Table 4 lists the articles with the highest impact factor. The paper, “Herb-drug interactions,” published in *The Lancet* in 2000 had the highest impact factor of over 47. The article, “In vitro flowering of embryoids derived from mature root callus of *ginseng* (*Panax-ginseng*),” published in *Nature* on 1980 had an impact factor of over 40.

Table 5 lists the top 10 references with the most citations from 1959 to 2016. Most of the top 10 cited references are reviews, and half of them were published before 2000.

## 4. Discussion

The number of research on *P. ginseng* worldwide showed a high growth between 1959 and 2016 and surged since 2000. Asia, especially South Korea and China, are the most active countries on *P. ginseng* research because this herb is mainly distributed in these countries (South Korea, 57.4%; and China, 31.1%). The amounts of *P. ginseng* produced in China and South Korea account for more than 55% and 34% of the total world output [1]. The top 10 organizations funding *P. ginseng* research all originate from South Korea and China. Hence, these two countries provide stronger financial support for *P. ginseng* research than the other countries. However, Japan greatly contributed to study of *P. ginseng* during 1973–2001. Saudi Arabia showed great interest in *P. ginseng* research since 2013, and most of the publications from this country are related to ginseng extracts.

The global cooperation pattern of different institutions is as important as the research output. Generally, institutions in South Korea and China have the highest number of publications and closest cooperation worldwide. Institutions from Japan, Russia, and UK exhibit less cooperation with the other countries. The USA belongs to the top publishing country but does not appear in the cooperation network (Figure 3). This finding suggests that research organizations in the USA are scattered.

Korean scholars conducted numerous studies on *P. ginseng* and published 41% of the total articles obtained. Korean authors, namely, KIM DH and KIM SH, submitted many publications and had high citations. In particular, KIM SH had burst during 2009–2016 and will become an important scholar in the *P. ginseng* domain in the near future.

China ranks second in terms of the quantity of publications. However, no institution on China belonged to the top 10 highest publishing journals. China still needs to improve the quality of *P. ginseng* research. China has abundant resources, a large number of research funding support, and high cooperativeness with other countries and thus exhibits high potential on *P. ginseng* research.

Figure 5a displays the main disciplines which are involved in *P. ginseng* research. Pharmacology, pharmacy, plant sciences, chemistry, integrative & complementary, biochemistry and molecular biology are the leading disciplines. Chemistry is the core discipline, which connects many other disciplines. Plant sciences are closely linked to integrative & complementary medicine, which have close cooperation with chemistry and medicine. Interestingly, chemistry is a bridge between these important subjects, like pharmacology & pharmacy, chemistry and medicinal. Figure 5b shows that the interest of scholars gradually changed from basic science to applied science during 1959–2016. Studies on neuroscience and neurology showed strong burst and have been the hotspots since 2000. At the bottom of Figure 5b, the subject categories of multidisciplinary sciences and science and technology exhibited a period of burst between 2014 and 2016, with burst strengths of over 7.6. This finding reveals a new trend of multidisciplinary disciplines in *P. ginseng* research. According to the clustering results of topics, numerous scientists focused on ginseng root, extracts, and cells that including cell activity, induction of ginseng cells and treatment of cells. At a fine-grained level, keywords with high frequency of occurrence indicate hotspots, such as ginsenoside, rat, cell, extract, expression, in vitro, and apoptosis. Inflammation, Alzheimer’s disease, compound k, and metabolite have the strongest burst since 2000. Therefore, these keywords will be the central concern of *P. ginseng* research in the near future. By analyzing disciplines, topics and key words, it's not difficult to find that the future study of *P. ginseng* may be biased towards clinical research and application, such as clinical neurology, toxicology and polymer science. Ginseng root, extracts (especially ginsenoside) and treatment of inflammation, Alzheimer’s disease would continue to be the research hotspots.

## 5. Conclusions

We utilized the visualization software CiteSpace to analyze the bibliographic data collected from the Web of Science^TM^ Core Collection database of *P. ginseng* research during 1959–2016. The research output showed a steady growth, and Asia, especially South Korea and China, was the most active area. A close collaboration was found between these countries. Our study reveals the intellectual landscape and detects emerging topics and trends. This study can help people who are unfamiliar with the active area of *P. ginseng* research to elucidate the global situation and overall structure of this domain. This paper also provides research hotspots, structured knowledge, and emerging trends with regard to *P. ginseng* research.

## Figures and Tables

**Table 1:** Top 10 highest publishing and sponsoring institutions in *P. ginseng* domain.

Rank	Research Institution	Publication Amount	Sponsor Institution	Sponsoring Amount
1	Kyung Hee University	323	National Natural Science Foundation of China	263
2	Seoul Natl University	198	Ministry of Education Science and Technology	73
3	Konkuk University	145	Korean Society of Ginseng	61
4	Chungbuk National University	135	Rural Development Administration Republic of Korea	37
5	Chinese Academy of Sciences	124	Korea ginseng corporation	37
6	Chungnam National University	108	Ministry of Education Science and Technology Republic of Korea	35
7	Kangwon National University	84	National Research Foundation of Korea Nrf	32
8	Jilin University	82	National Research Foundation of Korea	25
9	Korea University	75	Ministry of Science and Technology of China	22
10	Chonnam National University	69	Fundamental Research Funds for the Central Universities	22

**Table 2:** Languages of *P. ginseng* publication.

Rank	Language	Number	Percentage
1	English	3896	98.03
2	Chinese	34	0.86
3	Japanese	16	0.40
4	Russian	11	0.28
5	Portuguese	4	0.10
6	German	3	0.08
7	Polish	3	0.08
8	French	2	0.05
9	Hungarian	1	0.03
10	Italian	1	0.03
11	Korean	1	0.03
12	Spanish	1	0.03
13	Turkish	1	0.03

**Table 3:** The information about top 20 highest publishing journals.

Rank	Journal	Number	Percentage	IF	Region
1	Journal of Ginseng Research	313	7.874	4.082	South Korea
2	Journal of Ethnopharmacology	129	3.245	2.981	Ireland
3	Planta Medica	124	3.119	2.342	Germany
4	Biological & Pharmaceutical Bulletin	95	2.390	1.683	Japan
5	Phytotherapy Research	69	1.736	3.092	England
6	Food Science and Biotechnology	53	1.333	0.699	South Korea
7	American Journal of Chinese Medicine	53	1.333	3.222	USA
8	Archives of Pharmacal Research	52	1.308	2.324	South Korea
9	Chemical & Pharmaceutical Bulletin	51	1.283	1.133	Japan
10	Phytochemistry	45	1.132	3.205	England
11	Journal of Agricultural and Food Chemistry	45	1.132	3.154	USA
12	Evidence-Based Complementary and Alternative Medicine	43	1.082	1.740	England
13	Plant Cell Reports	40	1.006	2.869	Germany
14	Journal of Pharmaceutical and Biomedical Analysis	37	0.931	3.255	Netherlands
15	PLoS ONE	35	0.881	2.806	USA
16	Molecules	30	0.755	2.861	Switzerland
17	Plant Cell Tissue and Organ Culture	29	0.730	2.002	Netherlands
18	Phytomedicine	28	0.704	3.526	Germany
19	Journal of Chromatography A	26	0.654	3.981	Netherlands
20	Journal of Medicinal Food	24	0.604	1.955	South Korea

**Table 4:** Top 10 articles with the highest IF.

Rank	Title	Year	Journal	IF	Reference
1	Herb-drug interactions	2000	The Lancet	47.831	[12]
2	In vitro flowering of embryoids derived from mature root callus of *ginseng* (*Panax ginseng*)	1980	Nature	40.137	[13]
3	High-dose Asian ginseng (*Panax ginseng*) for cancer-related fatigue (CRF): A preliminary report	2013	Journal of Clinical Oncology	24.008	[14]
4	Herbal remedies in the United States: Potential adverse interactions with anticancer agents	2004	Journal of Clinical Oncology	24.008	[15]
5	Electrocardiographic and blood pressure effects of energy drinks and *Panax ginseng* in healthy volunteers: A randomized clinical trial	2016	Circulation	19.309	[16]
6	Progesterone regulates cardiac repolarization through a nongenomic pathway—An in vitro patch-clamp and computational modeling study	2007	Circulation	19.309	[17]
7	Modulating angiogenesis: the yin and the yang in ginseng	2004	Circulation	19.309	[18]
8	Production of bioactive ginsenoside compound K in metabolically engineered yeast	2014	Cell Research	15.606	[19]
9	Traditional Chinese medicine: an approach to scientific proof and clinical validation	2000	Pharmacology & Therapeutics	11.127	[20]
10	Enantioselective prophenol-catalyzed addition of 1,3-diynes to aldehydes to generate synthetically versatile building blocks and diyne natural products	2010	Journal of the American Chemical Society	13.858	[21]

**Table 5:** Top-10 most cited references.

Rank	Title	TLCS	Year	Journal	Reference
1	*Ginseng* pharmacology—Multiple constituents and multiple actions	512	1999	Biochemical Pharmacology	[22]
2	*Panax ginseng* pharmacology: A nitric oxide link?	232	1997	Biochemical Pharmacology.	[23]
3	Antidiabetic effects of *Panax ginseng* berry extract and the identification of an effective component	137	2002	Diabetes	[24]
4	Botanical characteristics, pharmacological effects and medicinal components of Korean *Panax ginseng* C. A. Meyer	134	2008	Acta Pharmacologica Sinica	[25]
5	Inhibitory effect of tumor-metastasis in mice by saponins, ginsenoside-rb2, 20(*r*)-ginsenoside-rg3 and 20(*s*)-ginsenoside-rg3, of red-ginseng	133	1995	Biological & Pharmaceutical Bulletin.	[26]
6	Recent advances on *ginseng* research in China	131	1992	Journal of Ethnopharmacology	[27]
7	Antioxidant and anti-tumor promoting activities of the methanol extract of heat-processed *ginseng*	116	2000	Cancer Letters.	[28]
8	*Panax ginseng*	115	2003	American Family Physician	[29]
9	Inhibition of tumor angiogenesis and metastasis by a saponin of *panax-ginseng*, ginsenoside-rb2	105	1994	Biological & Pharmaceutical Bulletin	[30]
10	Chemistry and cancer preventing activities of *ginseng* saponins and some related triterpenoid compounds	101	2001	Journal of Korean Medical Science.	[31]
